# Curve Effect on Singlet Diradical Contribution in Kekulé-type Diradicals: A Sensitive Probe for Quinoidal Structure in Curved π-Conjugated Molecules

**DOI:** 10.3390/molecules24010209

**Published:** 2019-01-08

**Authors:** Misaki Matsumoto, Ivana Antol, Manabu Abe

**Affiliations:** 1 Department of Chemistry, Graduate School of Science, Hiroshima University, 1-3-1 Kagamiyama, Higashi-Hiroshima, Hiroshima 739-8526, Japan; 36664u@ube-ind.co.jp; 2 Laboratory for Physical Organic Chemistry, Division of Organic Chemistry and Biochemistry, Ruđer Bošković Institute, Bijenička cesta 54, 10000 Zagreb, Croatia; 3 Hiroshima University Research Center for Photo-Drug-Delivery Systems (HiU-P-DDS), Hiroshima University, Hiroshima 739-8526, Japan; 4 JST-CREST, K’s Gobancho 6F, 7, Gobancho, Chiyoda-ku, Tokyo 102-0075, Japan

**Keywords:** Kekulé-type diradicals, curve effect, π-conjugated molecules, quinoidal structure, CASPT2/CASSCF calculations

## Abstract

Curved (non-planar) aromatic compounds have attracted significant research attention in the fields of basic chemistry and materials science. The contribution of the quinoidal structure in the curved π-conjugated structures has been proposed to be the key for materials functions. In this study, the curve effect on the quinoidal contribution was investigated in Kekulé-type singlet diradicals (S-**DR1-4**) as a sensitive probe for quinoidal structures in curved π-conjugated molecules. The quinoidal contribution in S-**DR1-4** was found to increase with increasing the curvature of the curved structure, which was quantitatively analyzed using NBO analysis and the natural orbital occupation numbers computed by the CASSCF method. The curve effect on the singlet-triplet energy gap was examined by the CASPT2 method. The singlet-triplet energy gaps for the highly π-conjugated diradicals were determined for the first time using the CASPT2 method. Substantial quinoidal contribution was found in the curved structures of the delocalized singlet diradicals S-**DR1-4**, in contrast to its absence in the corresponding triplet states T-**DR1-4**.

## 1. Introduction

**Figure 1:**
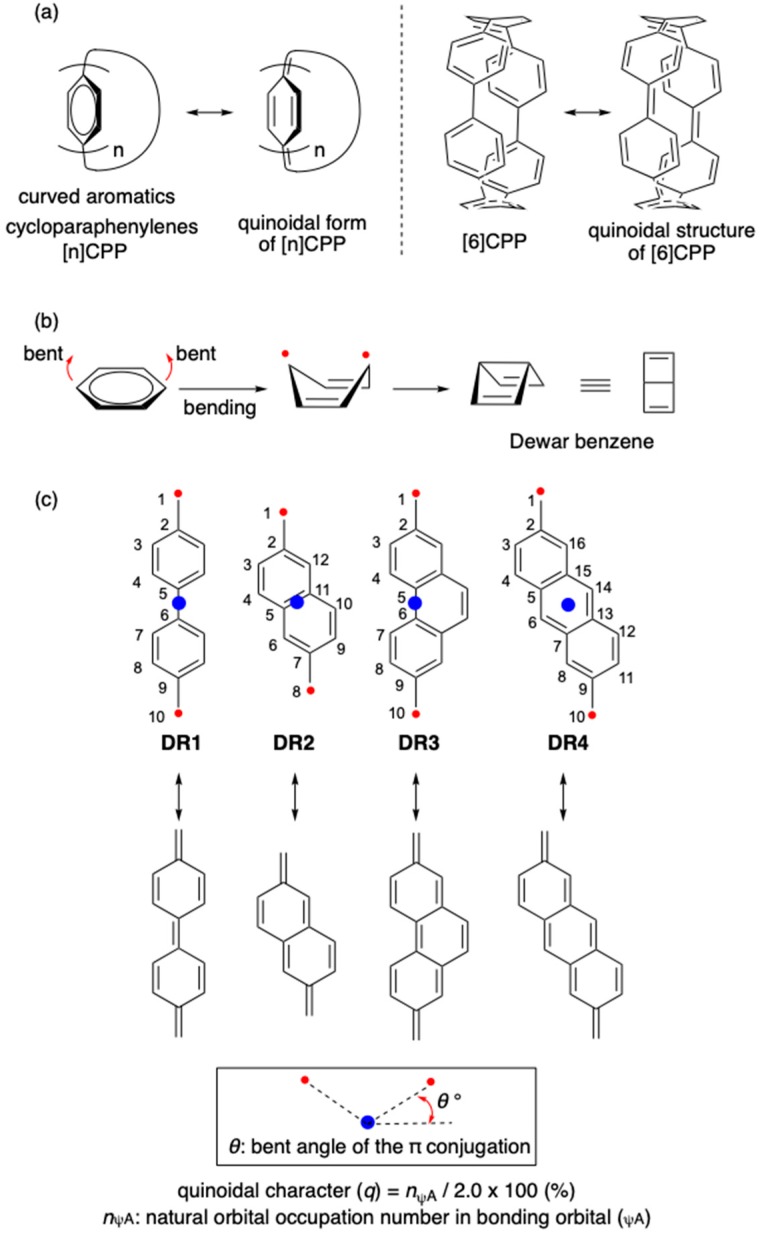
(**a**) [n]CPPs and their quinoidal structures; (**b**) bent effect on the diradical character of benzene [7]; (**c**) curve (*θ*°) effect on the diradical character in **DR1-4** as a sensitive probe for the quinoidal contribution (this study). The structures **DR1**, **DR2**, and **DR4** were optimized in *C*_2_ symmetry. The structure **DR3** was optimized in *C*_S_ symmetry.

Curved (non-planar) aromatic compounds like fullerenes, buckybowls, and carbon nanotubes have attracted considerable attention from researchers in the fields of basic chemistry as well as materials science [1,2,3,4,5,6,7,8,9,10,11]. In general, the HOMO–LUMO energy gap of a π-conjugated molecule decreases with increasing π-conjugation, leading to red-shifted absorption spectra [12]. However, according to recent reports on cycloparaphenylenes ([n]CPPs, n being the number of benzene rings in the structure) [13,14,15,16,17,18,19], which are hoop-shaped carbon molecules, the absorption bands were blue-shifted with increasing number of benzene rings [20,21,22,23,24,25]. This has been explained by the quinoidal characteristic of CPPs having small ring size, such as [6]CPP (Figure 1a). The quinoidal character of small CPPs has been proved by Raman spectroscopic analyses [26] and size-dependent-change of emission in CPPs [27,28]. 

The quinoidal contribution is rationalized by the increase of diradical character in the bent structure of benzene, which is the intermediate structure for the formation of Dewar benzene [29,30,31,32] (Figure 1b). In this study, the curve effect on the quinoidal character in Kekulé-type singlet diradicals S-**DR1-4** [33,34,35,36,37] is investigated to design a sensitive probe for the quinoidal contribution (*q*) in curved π-conjugated molecules (Figure 1c) [35,36,38,39,40,41,42,43,44,45,46,47,48,49,50,51,52,53,54,55].

## 2. Results and Discussion

### 2.1. Computations for DR1

**Figure 2:**
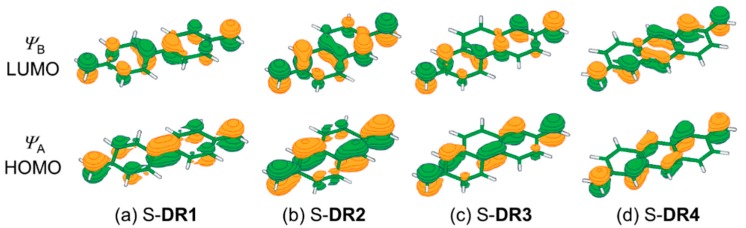
The molecular orbitals *ψ*_A_ (HOMO) and *ψ*_B_ (LUMO) for (**a**) S-**DR1**, (**b**) S-**DR2**, (**c**) S-**DR3**, and (**d**) S-**DR4**.

First, the curve effect on the diradical character was investigated on the 4,4′-dimethyl-1,1′-biphenyl-4,4′-diyl diradical (**DR1**) (Table 1, entries 1–4). The molecular structures of the singlet (S) and triplet (T) forms of **DR1** were optimized to obtain *C*_2_ symmetry at the UB3LYP/6-31G(d) level of theory with Gaussian 09 (revision D.01) software (Gaussian, Inc., Wallingford, CT, USA). The broken-symmetry (BS) method [56] was used for the optimization of S-**DR1** (See Supplementary Materials). The natural occupation numbers in the active orbitals were determined by the complete active-space multiconfiguration method at the CASSCF(14,14) [57]/cc-pVDZ [58] level of theory with MOLCAS 8 program package (v8.0.15-06-18) (MOLCAS, Lund, Sweden). The occupation number in orbital *ψ*_A_ (HOMO in the restricted Hartree-Fock (RHF) method) increased from 1.66 (*θ* = 0°, entry 1) to 1.80 (*θ* = 29°, entry 4) with increasing angle of bend (*θ*) in the diradical structures. The bent angles (*θ*) were obtained after the structural optimization of **DR1** in *C*_2_ symmetry at the fixed angles of C1–C2–C6 = C10–C9–C5 = 180, 160, 140, and 135°, respectively. On the other hand, the occupation number in orbital *ψ*_B_ (LUMO in the RHF method) decreased from 0.35 (*θ* = 0°, entry 1) to 0.21 (*θ* = 29°, entry 4) with increasing *θ*. Figure 2 shows that the HOMO and LUMO orbitals correspond to the bonding and anti-bonding orbitals of the quinoid form, respectively. The quinoidal contribution (*q*) was given by *q* (%) = (*n_ψ_*_A_/2.0) × 100, *n_ψ_*_A_ being the number of electrons in the HOMO orbital. The q values increased from 83 to 90 with increasing *θ*. The *θ*-dependent changes in the occupation number and *q* value indicates that the bonding interaction between the two phenyl-rings, leading to the formation of the quinoidal structure, increased with increasing *θ*. In fact, the C5–C6 distance was found to decrease from 143.0 pm (*θ* = 0°) to 139.6 pm (*θ* = 29°). The increase in quinoidal contribution was also proved by the decrease in C1–C2 bond distance and the increase in the Wiberg bond order (BO) [59] (entries 1–4). The computational results clearly indicate that the quinoidal contribution increases with increasing the extent of bending in S-**DR1**. 

The electronic energies of singlet ground state (1^1^A) S- and triplet (1^3^B) T-**DR1** were computed using the complete active-space second-order multiconfigurational perturbation theory (CASPT2) [61,62], including the dynamic corrections and cc-pVDZ basis set (Table 1). Active-space CAS (14,14) encompasses all π orbitals. The MO plots and weights of the leading configurations of the CASSCF wavefunction are provided in the Supporting Information. Both S- and T-**DR1** were destabilized with increasing *θ*, as reflected by the Δ*E*_rel,S_/Δ*E*_rel,__T_ values in Table 1. The relative energies, Δ*E*_rel,S_ and Δ*E*_rel,T_, were calculated with respect to the singlet and triplet absolute energies, respectively, in the planar structure (*θ* = 0°). The singlet–triplet energy difference, Δ*E*_ST_ = *E*_S_ − *E*_T_, increased substantially from 11.6 (*θ* = 0°, entry 1) to 19.3 kcal mol^–1^ (*θ* = 29°, entry 4). As judged by the curve effect on the electronic energies of S-**DR1** and T-**DR1**, i.e., Δ*E*_rel,S_/Δ*E*_rel,T_, the triplet state was destabilized more significantly than the singlet one with increasing *θ*. For example, at *θ* = 29° Δ*E*_rel,S_ and Δ*E*_rel,T_ were calculated to be +21.0 and +28.7 kcal mol^–1^, respectively (entry 4). The curve effect on Δ*E*_ST_ is rationalized by the quinoidal contribution to the curved structure of the singlet state S-**DR1**, which stabilized the singlet state, but not the triplet state. 

**Figure 3:**
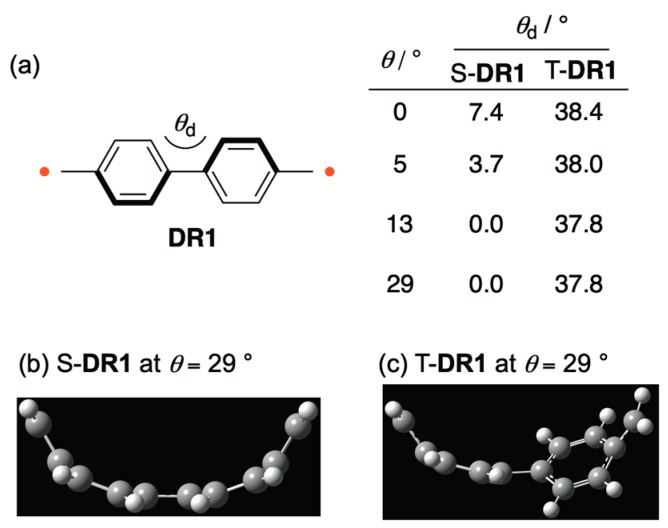
(**a**) The curve effect on the dihedral angle (*θ*_d_ = C4–C5–C6–C7) in **DR1**; (**b**) the optimized structure of S-**DR1** at *θ* = 29°; (**c**) the optimized structure of T-**DR1** at *θ* = 29°.

The quinoidal contribution in S-**DR1** was also rationalized by the curve effect on the dihedral angle (*θ*_d_ = C4–C5–C6–C7) of the biphenyl moiety (Figure 3). The dihedral angle *θ*_d_ decreased to nearly 0° from 7.4° when the bent angle *θ* increased from 0° to 29° (Figure 3a,b). In contrast to the significant curve effect on *θ*_d_, the corresponding dihedral angle was nearly the same in T-**DR1** because there was no quinoidal contribution in the triplet state (Figure 3c). 

### 2.2. Computations for **DR2-4**

Similar computations were conducted for the π-extended Kekulé-type diradicals, viz., 2,6-dimethylnaphthalene-2,6-diyl diradical (**DR2**) (entries 5–8), 2,7-dimethylphenanthrene-2,7-diyl diradical (**DR3**) (entries 9–12), and 2,6-dimethylanthracene-2,6-diyl diradical (**DR4**) (entries 13–16) to analyze the quinoidal contribution to the curved singlet states. As found for **DR1**, the quinoidal contribution (*q*) to the singlet state of **DR2-4** increased with increasing *θ* (entries 5–16). For **DR2**, the *q* value increased from 88.5 to 92.5 with increasing *θ*. The *q* values for other diradicals DR3 and DR4 were also found to increase with increasing their curved character: from 79 to 93.5 for S-**DR3** and from 82.5 to 91.0 for S-**DR4**. Consistent with the increase in quinoidal contribution, the C1–C2 bond in the singlet state became shorter than that in the triplet state, indicating that the quinoidal contribution to the singlet state increases with increasing *θ*. Furthermore, the Wiberg BOs of C1–C2 increased with increasing *θ* in **DR2-4**: from 1.68 to 1.76 for S-**DR2**, from 1.47 to 1.75 for S-**DR3**, and from 1.54 to 1.73 for S-**DR4**. As for **DR1**, the significant curve effect on the singlet–triplet energy gap (Δ*E*_ST_) was also computed for **DR2-4**. The energy gap increased with increasing *θ*, because the destabilization with increasing *θ* in the triplet states T-**DR2-4** were larger than those in the corresponding singlet states. 

## 3. Conclusions

In this study, the quinoidal contributions in curved aromatic structures were quantitatively analyzed by computing the curve effect on the diradical character of **DR1-4** at high-level ab initio calculations using the CASPT2/CASSCF method. The singlet-triplet energy gaps for the highly π-conjugated diradicals were determined for the first time using the CASPT2 method. The diradical character in the singlet states decreased with increasing the curve angle (*θ*) of the aromatic ring. In other words, the quinoidal contribution increases with increasing *θ* of the aromatic ring. The increases in the quinoidal contribution in the curved diradicals are consistent with the curve effect on the quinoidal character of hoop-shaped molecules, which has been intensively investigated in the last decade. The curved structure can increase the π-conjugation length with decreasing the HOMO-LUMO gap, which should be smaller than that in the planar molecules having the same number of π-electrons. The molecular design is expected to be appropriate for future soft-materials.

## Figures and Tables

**Table 1:** Curve effect, in terms of *θ* (°), on the natural occupation numbers in *ψ*_A_ (HOMO) and *ψ*_B_ (LUMO), quinoidal contribution (*q*), Wiberg bond order (BO), C1–C2 distance (pm), and singlet-triplet energy gap (Δ*E*_ST_, kcal mol^–1^) ^a^.

Entry	DR	Bent Angle *θ* (°)	Occupation Number		*q*	BO ^b^	C1–C2 Singlet/Triplet	Δ*E*_ST_^c^ Δ*E*_rel,S_/Δ*E*_rel,T_
			*ψ*_A_ (HOMO)	*ψ*_B_ (LUMO)				
1	**DR1**	0 (C1-C2-C6 = C10-C9-C5 = 180°)	1.66	0.35	83.0	1.55	137.4/140.8	11.6 0.0/0.0
2		13 (160°)	1.70	0.31	85.0	1.61	136.7/140.8	12.9 +4.4/+5.7
3		25 (140°)	1.79	0.23	89.5	1.73	135.6/140.4	17.4 +17.0/+22.8
4		29 (135°)	1.80	0.21	90.0	1.75	135.5/140.2	19.3 +21.0/+28.7
5	**DR2**	0 (C1-C2-C6 = C8-C7-C12 = 180°)	1.77	0.24	88.5	1.68	135.9/142.0	22.0 0.0/0.0
6		12 (160°)	1.78	0.23	89.0	1.69	135.8/142.0	23.3 +4.0/+5.2
7		17 (140°)	1.82	0.19	91.0	1.71	135.6/141.9	27.4 +15.4/+20.7
8		26 (120°)	1.85	0.16	92.5	1.76	135.2/141.6	35.5 +32.4/+45.8
9	**DR3**	0 (C1-C5-C6 = C10-C6-C5 = 180°)	1.58	0.43	79.0	1.47	138.5/141.2	11.8 +0.0/+0.0
10		17 (160°)	1.62	0.39	81.0	1.51	138.1/141.1	12.9 +5.9/+7.0
11		34 (140°)	1.76	0.25	88.0	1.67	136.2/140.8	16.6 +23.1/+27.9
12		52 (120 °)	1.87	0.14	93.5	1.75	135.6/140.2	26.7 +46.6/+61.4
13	**DR4**	0 (C1-C5-C6 = C10-C13-C14 = 180°)	1.65	0.36	82.5	1.54	137.5/140.6	15.2 +0.0/+0.0
14		12 (160°)	1.68	0.33	84.0	1.57	137.2/140.5	16.0 +5.0/+5.8
15		24 (140°)	1.76	0.25	88.0	1.68	136.0/140.3	18.3 +20.1/+23.2
16		35 (120°)	1.82	0.19	91.0	1.73	135.6/140.0	24.0 +43.0/+51.7

*^a^* The structural optimization was performed in *C*_2_ (**DR1**, **DR2** and **DR4**) and *C*_S_ (**DR3**) symmetry at the (U)B3LYP/6-31G(d) level of theory. The occupation numbers in *ψ*_A_ and *ψ*_B_ were computed at the CASCF/cc-pVDZ level of theory, CASSCF(14,14) for **DR1**, CASSCF(12,12) for **DR2**, and CASSCF(16,16) for **DR3** and **DR4**. The energies were obtained at the CASPT2/cc-pVDZ level of theory. *^b^* The Wiberg BO between C1 and C2 was determined by natural atomic orbital (NAO) and natural bond orbital (NBO) analyses at the B3LYP/6-31G(d) level of theory [60]. *^c^* The singlet-triplet energy gap, (Δ*E*_ST_), was determined to be *E*_S_ − *E*_T_. The energies, Δ*E*_rel,S_/Δ*E*_rel,T_, were relative to the absolute energy for *θ* = 0°.

## Supplementary Materials

The following are available online.
