# Supplementary material for: Curve Effect on Singlet Diradical Contribution in Kekulé-type Diradicals: A Sensitive Probe for Quinoidal Structure in Curved π-Conjugated Molecules
Source: Molecules. 2019 Jan 8;24(1):209. doi: 10.3390/molecules24010209 (PMC6337420; doi:10.3390/molecules24010209)
Supplement: Supplementary file 1 [file molecules-24-00209-s001.pdf]

Supporting information

Curve Effect on Singlet Diradical Contribution in Kekulé-type Diradicals. A Sensitive Probe for Quinoidal Structure in Curved π-Conjugated Molecules

Misaki Matsumoto[1], Ivana Antol*[,2] and Manabu Abe*[,1,3]

[1]Department of Chemistry, Graduate School of Science, Hiroshima University, 1-3-1 Kagamiyama, Higashi-Hiroshima, Hiroshima 739-8526, Japan

[2]Laboratory for Physical Organic Chemistry, Division of Organic Chemistry and Biochemistry, Ruđer Bošković Institute, Bijenička cesta 54, 10000 Zagreb, Croatia

[3]Hiroshima University Research Center for Photo-Drug-Delivery Systems (HiU-P-DDS)

*Correspondence: Ivana.Antol@irb.hr; mabe@hiroshima-u.ac.jp

Contents:

1. Active orbitals for S-**DR1** structure at θ = 0 ° (Figure S1)
2. Natural orbital occupation numbers for the S_0 state of S-**DR1** structures at different bent angles (Table S1)
3. Natural orbital occupation numbers for the T_1 state of T-**DR1** structures at different bent angles (Table S2)
4. Weights of the leading configurations calculated by the CASSCF method for ground state singlet (1^1A) state and triplet (1^3B) state of **DR1** structures with different bent angles (Table S3)
5. Active orbitals for S-**DR2** structure at θ = 0 ° (Figure S2)
6. Natural orbital occupation numbers for the S_0 state of S-**DR2** structures at different bent angles (Table S4)
7. Natural orbital occupation numbers for the T_1 state of T-**DR2** structures at different bent angles (Table S5)
8. Weights of the leading configurations calculated by the CASSCF method for ground state singlet (1^1A) state and triplet (1^3B) state of **DR2** structures with different bent angles (Table S6)
9. Active orbitals for S-**DR3** structure at θ = 0 ° (Figure S3)
10. Natural orbital occupation numbers for the S_0 state of S-**DR3** structures at different bent angles (Table S7)
11. Natural orbital occupation numbers for the T_1 state of T-**DR3** structures at different bent angles (Table S8)
12. Weights of the leading configurations calculated by the CASSCF method for ground state singlet (1^1A') state and triplet (1^3A'') state of **DR3** structures with different bent angles (Table S9)
13. Active orbitals for S-**DR4** structure at θ = 0 ° (Figure S4)
14. Natural orbital occupation numbers for the S_0 state of S-**DR4** structures at different bent angles (Table S10)
15. Natural orbital occupation numbers for the T_1 state of T-**DR4** structures at different bent angles (Table S11)
16. Weights of the leading configurations calculated by the CASSCF method for ground state singlet (1^1A) state and triplet (1^3B) state of **DR4** structures with different bent angles (Table S12)
17. Cartesian coordinates for **DR1-4**

DFT calculations have been done by Gaussian 09 (Revision D.01). MOLCAS 8 program package (v8.0.15-06-18) was used for the CASSCF and CASPT2 calculations.

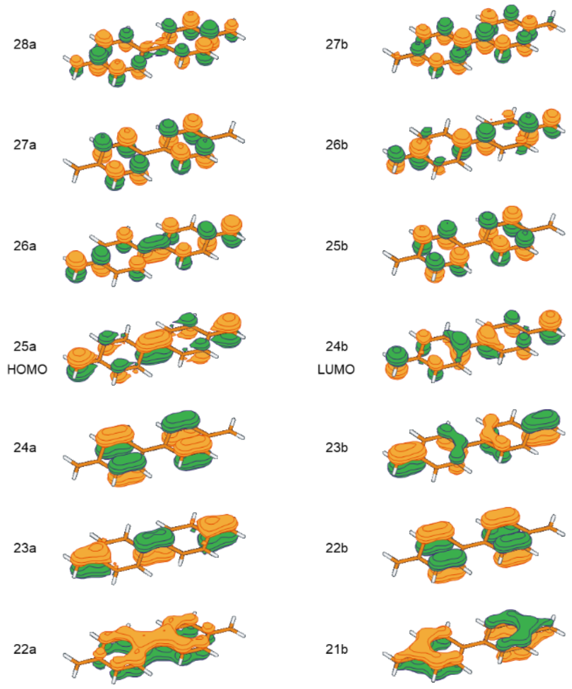

Figure S1. CASSCF active molecular orbitals for the S-**DR1** structure at $\theta = 0$ °. The calculation was calculated using the C_2 symmetry therefore the orbitals can be separated into a or b orbital symmetries.

Table S1. Natural orbital occupation numbers for active orbitals calculated for the S_0 state of S-**DR1** structures at different bent angles.

Orbital	Bent angle θ (C1-C2-C6 = C5-C9-C10) /°			
	0(180)	13(160)	25(140)	29(135)
22a	1.956	1.954	1.951	1.950
23a	1.922	1.922	1.920	1.918
24a	1.905	1.906	1.907	1.906
25a (HOMO, ψ_{A}.)	1.652	1.702	1.786	1.799
26a	0.130	0.126	0.116	0.114
27a	0.091	0.090	0.088	0.088
28a	0.047	0.049	0.055	0.058
21b	1.948	1.946	1.941	1.939
22b	1.908	1.909	1.911	1.911
23b	1.873	1.877	1.886	1.887
24b (LUMO, ψ_{B}.)	0.358	0.309	0.226	0.212
25b	0.096	0.095	0.093	0.093
26b	0.076	0.076	0.078	0.080
27b	0.039	0.040	0.044	0.045

Table S2. Natural orbital occupation numbers for active orbitals calculated for active orbitals calculated for the T_1 state of T-**DR1** structures at different bent angles

Orbital	Bent angle θ (C1-C2-C6 = C5-C9-C10) /°			
	0(180)	13(160)	25(140)	29(135)

22a	1.958	1.893	1.889	1.887
23a	1.903	1.957	1.954	1.952
24a	1.895	1.900	1.892	1.889
25a (SOMO, ψ_A.)	1.017	1.018	1.022	1.023
26a	0.120	0.108	0.111	0.112
27a	0.107	0.122	0.130	0.132
28a	0.042	0.044	0.049	0.050
21b	1.954	1.881	1.873	1.870
22b	1.894	1.953	1.949	1.948
23b	1.884	1.893	1.890	1.888
24b (SOMO, ψ_B.)	0.986	0.985	0.981	0.979
25b	0.107	0.109	0.113	0.114
26b	0.094	0.097	0.106	0.109
27b	0.038	0.039	0.043	0.044

Table S3. Weights (*w*) of the leading configurations calculated by the CASSCF method for ground state singlet (1^1A) state and triplet (1^3B) state of **DR1** structures with different bent angles

Bent angle θ (C1-C2-C6 = C5-C9-C10) /°	Singlet (1^1A, gr. state, S_0)		Triplet (1^3B, T_1)
	$w\ (\ldots(\psi_A)^2)$	$w\ (\ldots(\psi_B)^2)$	$w\ (\ldots(\psi_A)^1(\psi_B)^1)$
0 (180)	0.63599	0.08384	0.71232
13 (160)	0.65805	0.06517	0.70539
25 (140)	0.69408	0.0356	0.68387
29 (135)	0.69866	0.0311	0.6763

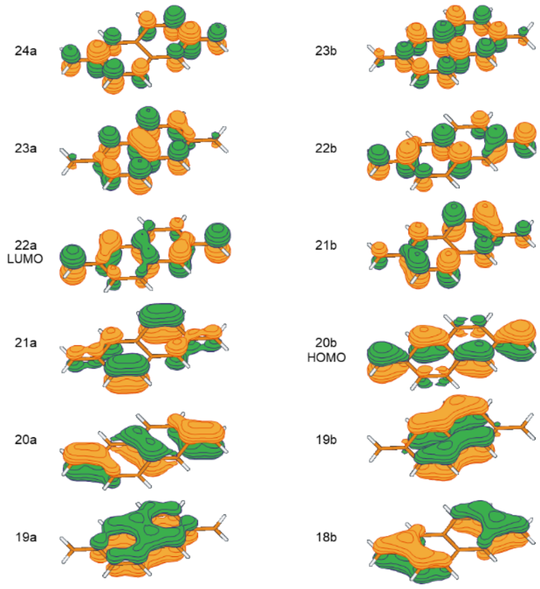

Figure S2. CASSCF active molecular orbitals for the S-**DR2** structure at θ = 0 °.

Table S4. Natural orbital occupation numbers for active orbitals calculated for the S_0 state of S-**DR2** structures at different bent angles

Orbital	Bent angle θ (C1-C2-C6 = C8-C7-C12) /°			
	0 (180)	12 (160)	17 (140)	26 (120)
19a	1.956	1.954	1.951	1.946
20a	1.911	1.910	1.908	1.904
21a	1.884	1.885	1.888	1.892
22a (LUMO, ψ_B.)	0.241	0.228	0.195	0.157
23a	0.073	0.074	0.076	0.080
24a	0.055	0.057	0.063	0.071
18b	1.940	1.939	1.934	1.928
19b	1.925	1.925	1.922	1.917
20b (HOMO, ψ_A.)	1.770	1.783	1.816	1.852
21b	0.117	0.116	0.113	0.107
22b	0.089	0.089	0.091	0.094
23b	0.038	0.039	0.044	0.051

Table S5. Natural orbital occupation numbers for active orbitals calculated for the T_1 state of T-**DR2** structures at different bent angles

Orbital	Bent angle θ (C1-C2-C6 = C8-C7-C12) /°			
	0 (180)	17 (160)	34 (140)	52 (120)
19a	1.960	1.959	1.956	1.951
20a	1.894	1.891	1.881	1.863
21a	1.855	1.854	1.850	1.843
22a (SOMO, ψ_B.)	0.966	0.964	0.957	0.940
23a	0.088	0.090	0.095	0.108
24a	0.057	0.058	0.064	0.073
18b	1.940	1.939	1.934	1.927
19b	1.911	1.909	1.904	1.892
20b (SOMO, ψ_A.)	1.037	1.039	1.046	1.060
21b	0.150	0.151	0.153	0.158
22b	0.107	0.110	0.121	0.140
23b	0.035	0.036	0.039	0.046

Table S6. Weights (*w*) of the leading configurations calculated by the CASSCF method for ground state singlet (1^1A) state and triplet (1^3B) state of **DR2** structures with different bent angles

Bent angle θ (C1-C2-C6 = C8-C7-C12) /°	Singlet (1^1A, gr. state, S_0)		Triplet (1^3B, T_1)
	w (…(ψ_A)^2)	w (…(ψ_B)^2)	w (…(ψ_A)^1(ψ_B)^1)
0 (180)	0.72420	0.04872	0.74879
17 (160)	0.72546	0.04726	0.74337
34 (140)	0.72869	0.04317	0.7252
52 (120)	0.73358	0.03696	0.69058

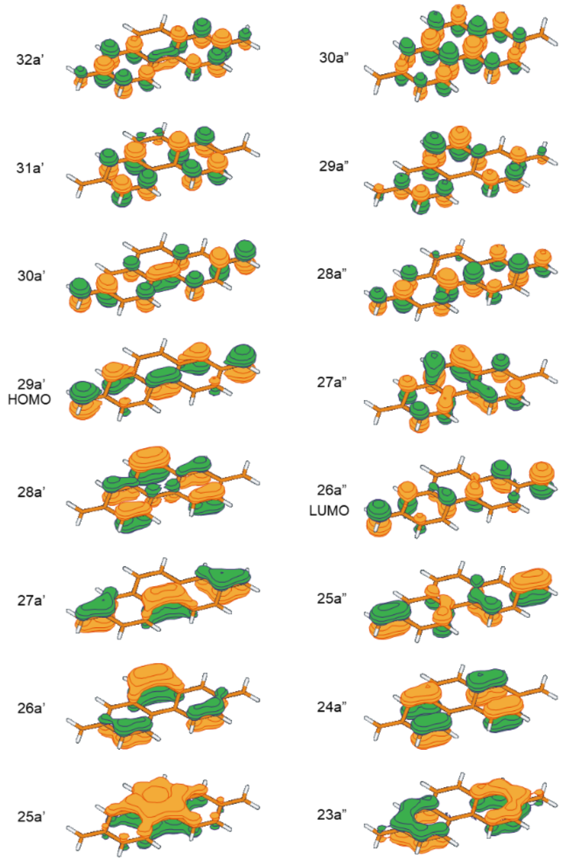

Figure S3. CASSCF active molecular orbitals for the S-**DR3** structure at $\theta = 0$ °. The calculation was calculated using the C_s symmetry therefore the orbitals can be separated into a' or a" orbital symmetries.

Table S7. Natural orbital occupation numbers for active orbitals calculated for the S_0 state of S-**DR3** structures at different bent angles

Orbital	Bent angle θ (C1-C5-C6 = C10-C6-C5) /°			
	0 (180)	17 (160)	34 (140)	52 (120)
25a'	1.960	1.958	1.953	1.994
26a'	1.935	1.934	1.931	1.939
27a'	1.922	1.921	1.919	1.927
28a'	1.868	1.871	1.883	1.901
29a' (HOMO, $\psi_{A\cdot}$)	1.580	1.622	1.759	1.868
30a'	0.128	0.127	0.118	0.108
31a'	0.091	0.092	0.091	0.089
32a '	0.046	0.048	0.051	0.056
23a"	1.949	1.948	1.944	1.940
24a"	1.909	1.909	1.909	1.908
25a"	1.874	1.875	1.883	1.893
26a" (LUMO, $\psi_{B\cdot}$)	0.429	0.388	0.254	0.142
27a"	0.134	0.131	0.117	0.098
28a"	0.077	0.078	0.079	0.071
29a"	0.062	0.063	0.066	0.059
30a"	0.034	0.036	0.043	0.006

Table S8. Natural orbital occupation numbers for active orbitals calculated for the T_1 state of T-**DR3** structures at different bent angles

Orbital	Bent angle θ (C1-C5-C6 = C10-C6-C5) /°			
	0 (180)	17 (160)	34 (140)	52 (120)
25a'	1.963	1.961	1.958	1.951
26a'	1.935	1.934	1.931	1.926
27a'	1.911	1.909	1.901	1.888
28a'	1.857	1.858	1.860	1.864
29a' (SOMO, ψ_A.)	1.027	1.028	1.032	1.039
30a'	0.125	0.127	0.132	0.141
31a'	0.100	0.102	0.107	0.117
32a '	0.044	0.045	0.048	0.054
23a"	1.952	1.952	1.949	1.942
24a"	1.901	1.899	1.894	1.885
25a"	1.879	1.877	1.869	1.856
26a" (SOMO, ψ_B.)	0.977	0.976	0.972	0.964
27a"	0.148	0.146	0.142	0.138
28a"	0.087	0.090	0.100	0.116
29a"	0.062	0.063	0.066	0.072
30a"	0.032	0.034	0.039	0.049

Table S9. Weights (*w*) of the leading configurations calculated by the CASSCF method for ground state singlet ($1^1A'$) state and triplet ($1^3A''$) state of **DR3** structures with different bent angles

Bent angle θ (C1-C5-C6 = C10-C6-C5) /°	Singlet ($1^1A'$, gr. state, S_0)		Triplet ($1^3A''$, T_1)
	$w(\ldots(\psi_A)^2)$	$w(\ldots(\psi_B)^2)$	$w(\ldots(\psi_A)^1(\psi_B)^1)$
0 (180)	0.5760	0.1048	0.68240
17 (160)	0.5926	0.0884	0.67713
34 (140)	0.6506	0.0396	0.65991
52 (120)	0.7238	-	0.62888

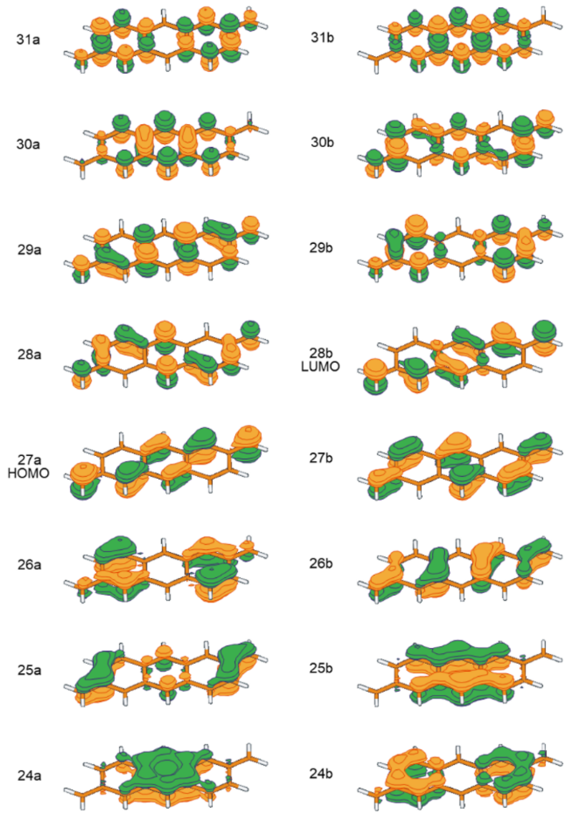

Figure S4. CASSCF active molecular orbitals for the S-**DR4** structure at θ = 0 °.

Table S10. Natural orbital occupation numbers for active orbitals calculated for the S_0 state of S-**DR4** structures at different bent angles

Orbital	Bent angle θ (C1-C5-C6 = C10-C13-C14) /°			
	0 (180)	12 (160)	24 (140)	35 (120)
24a	1.959	1.958	1.953	1.947
25a	1.930	1.930	1.929	1.925
26a	1.904	1.904	1.905	1.905
27a (HOMO, ψ_{A}.)	1.654	1.680	1.762	1.816
28a	0.159	0.155	0.140	0.127
29a	0.104	0.104	0.103	0.109
30a	0.070	0.071	0.074	0.078
31a	0.046	0.047	0.050	0.055
24b	1.948	1.948	1.945	1.941
25b	1.929	1.928	1.925	1.918
26b	1.897	1.897	1.897	1.893
27b	1.844	1.848	1.861	1.872
28b (LUMO, ψ_{B}.)	0.357	0.332	0.251	0.195
29b	0.095	0.095	0.094	0.095
30b	0.067	0.067	0.068	0.072
31b	0.035	0.036	0.042	0.050

Table S11. Natural orbital occupation numbers for the T_1 state of T-**DR4** structures at different bent angles

Orbital	Bent angle θ (C1-C5-C6 = C10-C13-C14) /°			
	0 (180)	12 (160)	24 (140)	35 (120)
24a	1.963	1.962	1.959	1.953
25a	1.930	1.929	1.927	1.924
26a	1.892	1.891	1.888	1.883
27a (SOMO, ψ_A.)	1.035	1.036	1.041	1.050
28a	0.182	0.181	0.179	0.176
29a	0.112	0.115	0.123	0.142
30a	0.078	0.079	0.084	0.092
31a	0.046	0.047	0.051	0.057
24b	1.949	1.948	1.945	1.940
25b	1.922	1.920	1.916	1.908
26b	1.890	1.887	1.878	1.860
27b	1.825	1.825	1.825	1.826
28b (SOMO, ψ_B.)	0.969	0.967	0.963	0.953
29b	0.108	0.110	0.113	0.118
30b	0.068	0.069	0.071	0.074
31b	0.032	0.033	0.037	0.045

Table S12. Weights (*w*) of the leading configurations calculated by the CASSCF method for ground state singlet (1^1A) state and triplet (1^3B) state of **DR4** structures with different bent angles

Bent angle θ (C1-C5-C6 = C10-C13-C14) /°	Singlet (1^1A, gr. state, S_0)		Triplet (1^3B, T_1)
	w (…$(\psi_A)^2$)	w (…$(\psi_B)^2$)	w (…$(\psi_A)^1(\psi_B)^1$)
0 (180)	0.60455	0.07488	0.66384
12 (160)	0.61484	0.06542	0.65972
24 (140)	0.64938	0.03727	0.64668
35 (120)	0.66686	0.01884	0.62363

S-**DR1** at θ = 0 ° (C1-C2-C6 = C5-C9-C10 = 180 °)

Standard orientation:

Center Number	Atomic Number	Atomic Type	Coordinates (Angstroms) X	Y	Z
1	1	0	-0.161861	0.969752	2.164907
2	6	0	-0.078652	1.482307	1.213291
3	6	0	0.082629	2.847531	-1.218371
4	6	0	0.000000	0.715202	-0.000003
5	6	0	-0.082619	2.847527	1.218371
6	6	0	0.000000	3.616545	0.000001
7	6	0	0.078659	1.482311	-1.213296
8	1	0	-0.155369	3.383575	2.161662
9	1	0	0.161868	0.969759	-2.164912
10	1	0	0.155387	3.383583	-2.161660
11	6	0	0.000000	-0.715202	-0.000003
12	6	0	0.000000	-3.616545	0.000001
13	6	0	0.078652	-1.482307	1.213291
14	6	0	-0.078659	-1.482311	-1.213296
15	6	0	-0.082629	-2.847531	-1.218371
16	6	0	0.082619	-2.847527	1.218371
17	1	0	0.161861	-0.969752	2.164907
18	1	0	-0.161868	-0.969759	-2.164912
19	1	0	-0.155387	-3.383583	-2.161660
20	1	0	0.155369	-3.383575	2.161662
21	6	0	-0.000008	-4.990477	0.000004
22	1	0	0.063355	-5.556354	0.924407
23	6	0	0.000008	4.990477	0.000004
24	1	0	0.063388	5.556360	-0.924394
25	1	0	-0.063355	5.556354	0.924407
26	1	0	-0.063388	-5.556360	-0.924394

T-**DR1** at θ = 0 ° (C1-C2-C6 = C5-C9-C10 = 180 °)

Standard orientation:

Center Number	Atomic Number	Atomic Type	Coordinates (Angstroms) X	Y	Z
1	1	0	-0.727358	2.025164	0.934885
2	6	0	-0.394406	1.140882	1.471467
3	6	0	0.395801	-1.146735	2.855700
4	6	0	0.000000	0.000000	0.741234
5	6	0	-0.395801	1.146735	2.855700
6	6	0	0.000000	0.000000	3.603213
7	6	0	0.394406	-1.140882	1.471467
8	1	0	-0.712728	2.038076	3.391724
9	1	0	0.727358	-2.025164	0.934885
10	1	0	0.712728	-2.038076	3.391724
11	6	0	0.000000	0.000000	-0.741234
12	6	0	0.000000	0.000000	-3.603213
13	6	0	0.394406	1.140882	-1.471467
14	6	0	-0.394406	-1.140882	-1.471467
15	6	0	-0.395801	-1.146735	-2.855700
16	6	0	0.395801	1.146735	-2.855700
17	1	0	0.727358	2.025164	-0.934885
18	1	0	-0.727358	-2.025164	-0.934885
19	1	0	-0.712728	-2.038076	-3.391724
20	1	0	0.712728	2.038076	-3.391724
21	6	0	0.000000	0.000000	-5.011499
22	1	0	0.299878	0.878412	-5.573516
23	6	0	0.000000	0.000000	5.011499
24	1	0	0.299878	-0.878412	5.573516
25	1	0	-0.299878	0.878412	5.573516
26	1	0	-0.299878	-0.878412	-5.573516

S-**DR1** at θ = 13 ° (C1-C2-C6 = C5-C9-C10 = 160 °)

Standard orientation:

Center Number	Atomic Number	Atomic Type	Coordinates (Angstroms) X	Y	Z
1	1	0	-2.172772	0.980219	0.396710
2	6	0	-1.218634	1.483358	0.288890
3	6	0	1.224332	2.829578	0.085619
4	6	0	0.000007	0.709970	0.317790
5	6	0	-1.224349	2.829553	0.085789
6	6	0	-0.000035	3.570832	-0.140239
7	6	0	1.218626	1.483420	0.288937
8	1	0	-2.169406	3.363617	0.021804
9	1	0	2.172776	0.980434	0.397394
10	1	0	2.169313	3.363755	0.021625
11	6	0	-0.000007	-0.709970	0.317790
12	6	0	0.000035	-3.570832	-0.140239
13	6	0	-1.218626	-1.483420	0.288937
14	6	0	1.218634	-1.483358	0.288890
15	6	0	1.224349	-2.829553	0.085789
16	6	0	-1.224332	-2.829578	0.085619
17	1	0	-2.172776	-0.980434	0.397394
18	1	0	2.172772	-0.980219	0.396710
19	1	0	2.169406	-3.363617	0.021804
20	1	0	-2.169313	-3.363755	0.021625
21	6	0	0.000007	-4.799619	-0.740138
22	1	0	-0.926361	-5.313623	-0.978755
23	6	0	-0.000007	4.799619	-0.740138
24	1	0	0.926361	5.313623	-0.978755
25	1	0	-0.926286	5.313812	-0.978670
26	1	0	0.926286	-5.313812	-0.978670

T-**DR1** at θ = 13 ° (C1-C2-C6 = C5-C9-C10 = 160 °)

Standard orientation:

Center Number	Atomic Number	Atomic Type	Coordinates (Angstroms) X	Y	Z
1	1	0	-1.964028	1.054021	1.045702
2	6	0	-1.074489	1.534844	0.647375
3	6	0	1.226981	2.779028	-0.315595
4	6	0	0.037207	0.740705	0.291944
5	6	0	-1.066697	2.907409	0.469589
6	6	0	0.061139	3.572089	-0.097987
7	6	0	1.205698	1.406686	-0.134965
8	1	0	-1.958659	3.486700	0.695792
9	1	0	2.083739	0.822932	-0.397413
10	1	0	2.117445	3.255808	-0.718120
11	6	0	-0.037207	-0.740705	0.291944
12	6	0	-0.061139	-3.572089	-0.097987
13	6	0	-1.205698	-1.406686	-0.134965
14	6	0	1.074489	-1.534844	0.647375
15	6	0	1.066697	-2.907409	0.469589
16	6	0	-1.226981	-2.779028	-0.315595
17	1	0	-2.083739	-0.822932	-0.397413
18	1	0	1.964028	-1.054021	1.045702
19	1	0	1.958659	-3.486700	0.695792
20	1	0	-2.117445	-3.255808	-0.718120
21	6	0	0.061139	-4.852228	-0.670152
22	1	0	-0.773625	-5.321748	-1.180522
23	6	0	-0.061139	4.852228	-0.670152
24	1	0	0.773625	5.321748	-1.180522
25	1	0	-0.982451	5.419666	-0.586700
26	1	0	0.982451	-5.419666	-0.586700

S-**DR1** at θ = 25 ° (C1-C2-C6 = C5-C9-C10 = 140 °)

Standard orientation:

Center Number	Atomic Number	Atomic Type	Coordinates (Angstroms) X	Y	Z
1	1	0	2.170654	1.009522	-0.805018
2	6	0	1.225658	1.483256	-0.561575
3	6	0	-1.231914	2.772395	-0.147689
4	6	0	-0.000041	0.699181	-0.614957
5	6	0	1.231837	2.772471	-0.147950
6	6	0	-0.000009	3.411304	0.314101
7	6	0	-1.225754	1.483166	-0.561297
8	1	0	2.170073	3.311650	-0.042033
9	1	0	-2.170801	1.009397	-0.804486
10	1	0	-2.170163	3.311501	-0.041504
11	6	0	0.000041	-0.699181	-0.614957
12	6	0	0.000009	-3.411304	0.314101
13	6	0	1.225754	-1.483166	-0.561297
14	6	0	-1.225658	-1.483256	-0.561575
15	6	0	-1.231837	-2.772471	-0.147950
16	6	0	1.231914	-2.772395	-0.147689
17	1	0	2.170801	-1.009397	-0.804486
18	1	0	-2.170654	-1.009522	-0.805018
19	1	0	-2.170073	-3.311650	-0.042033
20	1	0	2.170163	-3.311501	-0.041504
21	6	0	-0.000009	-4.234508	1.391478
22	1	0	0.925938	-4.596067	1.830221
23	6	0	0.000009	4.234508	1.391478
24	1	0	-0.925938	4.596067	1.830221
25	1	0	0.925938	4.596197	1.830154
26	1	0	-0.925938	-4.596197	1.830154

T-**DR1** at θ = 25 ° (C1-C2-C6 = C5-C9-C10 = 140 °)

Standard orientation:

Center Number	Atomic Number	Atomic Type	Coordinates (Angstroms) X	Y	Z
1	1	0	1.893686	1.180758	-1.335865
2	6	0	1.003130	1.596686	-0.871601
3	6	0	-1.308908	2.678754	0.251801
4	6	0	-0.074988	0.739014	-0.554438
5	6	0	0.982301	2.935636	-0.524242
6	6	0	-0.121753	3.479782	0.209643
7	6	0	-1.269163	1.340413	-0.098814
8	1	0	1.870967	3.545519	-0.668470
9	1	0	-2.139845	0.718192	0.089809
10	1	0	-2.199076	3.083187	0.727618
11	6	0	0.074988	-0.739014	-0.554438
12	6	0	0.121753	-3.479782	0.209643
13	6	0	1.269163	-1.340413	-0.098814
14	6	0	-1.003130	-1.596686	-0.871601
15	6	0	-0.982301	-2.935636	-0.524242
16	6	0	1.308908	-2.678754	0.251801
17	1	0	2.139845	-0.718192	0.089809
18	1	0	-1.893686	-1.180758	-1.335865
19	1	0	-1.870967	-3.545519	-0.668470
20	1	0	2.199076	-3.083187	0.727618
21	6	0	-0.121753	-4.400425	1.241462
22	1	0	0.666716	-4.700760	1.924812
23	6	0	0.121753	4.400425	1.241462
24	1	0	-0.666716	4.700760	1.924812
25	1	0	1.084151	4.893463	1.339232
26	1	0	-1.084151	-4.893463	1.339232

S-**DR1** at *θ* = 29 ° (C1-C2-C6 = C5-C9-C10 = 135 °)

Standard orientation:

Center Number	Atomic Number	Atomic Type	Coordinates (Angstroms) X	Y	Z
1	1	0	2.168252	1.021188	-0.897723
2	6	0	1.226460	1.483572	-0.621083
3	6	0	-1.233074	2.754373	-0.156009
4	6	0	-0.000054	0.698160	-0.681039
5	6	0	1.232972	2.754499	-0.156330
6	6	0	-0.000013	3.355818	0.358491
7	6	0	-1.226589	1.483442	-0.620778
8	1	0	2.168754	3.295723	-0.040214
9	1	0	-2.168449	1.021017	-0.897138
10	1	0	-2.168876	3.295500	-0.039588
11	6	0	0.000054	-0.698160	-0.681039
12	6	0	0.000013	-3.355818	0.358491
13	6	0	1.226589	-1.483442	-0.620778
14	6	0	-1.226460	-1.483572	-0.621083
15	6	0	-1.232972	-2.754499	-0.156330
16	6	0	1.233074	-2.754373	-0.156009
17	1	0	2.168449	-1.021017	-0.897138
18	1	0	-2.168252	-1.021188	-0.897723
19	1	0	-2.168754	-3.295723	-0.040214
20	1	0	2.168876	-3.295500	-0.039588
21	6	0	-0.000013	-4.048669	1.522786
22	1	0	0.925908	-4.359334	1.999256
23	6	0	0.000013	4.048669	1.522786
24	1	0	-0.925908	4.359334	1.999256
25	1	0	0.925915	4.359511	1.999183
26	1	0	-0.925915	-4.359511	1.999183

T-**DR1** at *θ* = 29 ° (C1-C2-C6 = C5-C9-C10 = 135 °)

Standard orientation:

Center Number	Atomic Number	Atomic Type	Coordinates (Angstroms) X	Y	Z
1	1	0	1.873128	1.214241	-1.403194
2	6	0	0.983612	1.612251	-0.921402
3	6	0	-1.330169	2.649798	0.241298
4	6	0	-0.084811	0.738371	-0.612638
5	6	0	0.959509	2.939837	-0.533951
6	6	0	-0.136837	3.446998	0.241445
7	6	0	-1.285163	1.323060	-0.149657
8	1	0	1.846022	3.556608	-0.661087
9	1	0	-2.153666	0.692335	0.020098
10	1	0	-2.220336	3.034918	0.732847
11	6	0	0.084811	-0.738371	-0.612638
12	6	0	0.136837	-3.446998	0.241445
13	6	0	1.285163	-1.323060	-0.149657
14	6	0	-0.983612	-1.612251	-0.921402
15	6	0	-0.959509	-2.939837	-0.533951
16	6	0	1.330169	-2.649798	0.241298
17	1	0	2.153666	-0.692335	0.020098
18	1	0	-1.873128	-1.214241	-1.403194
19	1	0	-1.846022	-3.556608	-0.661087
20	1	0	2.220336	-3.034918	0.732847
21	6	0	-0.136837	-4.249486	1.358681
22	1	0	0.639113	-4.497994	2.076622
23	6	0	0.136837	4.249486	1.358681
24	1	0	-0.639113	4.497994	2.076622
25	1	0	1.109123	4.714408	1.492056
26	1	0	-1.109123	-4.714408	1.492056

S-**DR2** at *θ* = 0 ° (C1-C2-C6 = C8-C7-C12 = 180 °)

Standard orientation:

Center Number	Atomic Number	Atomic Type	Coordinates (Angstroms) X	Y	Z
1	6	0	2.459561	0.790624	0.000000
2	1	0	1.132456	2.523908	0.000000
3	6	0	1.159491	1.435945	0.000000
4	6	0	1.310199	-1.380133	0.000000
5	6	0	-0.010893	0.732829	0.000000
6	6	0	2.459561	-0.670278	0.000000
7	6	0	0.010893	-0.732829	0.000000
8	6	0	-1.310199	1.380133	0.000000
9	1	0	3.421271	-1.177412	0.000000
10	1	0	-1.132456	-2.523908	0.000000
11	1	0	1.334335	-2.467627	0.000000
12	6	0	-2.459561	0.670278	0.000000
13	1	0	-1.334335	2.467627	0.000000
14	1	0	-3.421271	1.177412	0.000000
15	6	0	-2.459561	-0.790624	0.000000
16	6	0	-1.159491	-1.435945	0.000000
17	6	0	3.616365	1.503167	0.000000
18	1	0	4.585041	1.012437	0.000000
19	1	0	3.614174	2.589171	0.000000
20	6	0	-3.616365	-1.503167	0.000000
21	1	0	-4.585041	-1.012437	0.000000
22	1	0	-3.614174	-2.589171	0.000000

T-**DR2** at *θ* = 0 ° (C1-C2-C6 = C8-C7-C12 = 180 °)

Standard orientation:

Center Number	Atomic Number	Atomic Type	Coordinates (Angstroms) X	Y	Z
1	6	0	2.471428	0.718163	0.000000
2	1	0	1.242303	2.488396	0.000000
3	6	0	1.245648	1.400429	0.000000
4	6	0	1.245648	-1.401908	0.000000
5	6	0	0.004617	0.714655	0.000000
6	6	0	2.432674	-0.719413	0.000000
7	6	0	-0.004617	-0.714655	0.000000
8	6	0	-1.245648	1.401908	0.000000
9	1	0	3.373935	-1.263569	0.000000
10	1	0	-1.242303	-2.488396	0.000000
11	1	0	1.241130	-2.489559	0.000000
12	6	0	-2.432674	0.719413	0.000000
13	1	0	-1.241130	2.489559	0.000000
14	1	0	-3.373935	1.263569	0.000000
15	6	0	-2.471428	-0.718163	0.000000
16	6	0	-1.245648	-1.400429	0.000000
17	6	0	3.710892	1.410430	0.000000
18	1	0	4.653470	0.873486	0.000000
19	1	0	3.746319	2.494701	0.000000
20	6	0	-3.710892	-1.410430	0.000000
21	1	0	-4.653470	-0.873486	0.000000
22	1	0	-3.746319	-2.494701	0.000000

S-**DR2** at θ = 12 ° (C1-C2-C6 = C8-C7-C12 = 160 °)

Standard orientation:

Center Number	Atomic Number	Atomic Type	Coordinates (Angstroms) X	Y	Z
1	6	0	-1.824976	1.795778	0.055189
2	1	0	0.137776	2.755968	-0.029838
3	6	0	-0.378663	1.800008	-0.090429
4	6	0	-1.793722	-0.633533	-0.287775
5	6	0	0.342296	0.648826	-0.223860
6	6	0	-2.494751	0.514901	-0.169688
7	6	0	-0.342296	-0.648826	-0.223860
8	6	0	1.793722	0.633533	-0.287775
9	1	0	-3.581879	0.502470	-0.173320
10	1	0	-0.137776	-2.755968	-0.029838
11	1	0	-2.308225	-1.586110	-0.393274
12	6	0	2.494751	-0.514901	-0.169688
13	1	0	2.308225	1.586110	-0.393274
14	1	0	3.581879	-0.502470	-0.173320
15	6	0	1.824976	-1.795778	0.055189
16	6	0	0.378663	-1.800008	-0.090429
17	6	0	-2.494751	2.863013	0.561225
18	1	0	-3.567486	2.832795	0.728320
19	1	0	-1.985405	3.792082	0.800137
20	6	0	2.494751	-2.863013	0.561225
21	1	0	3.567486	-2.832795	0.728320
22	1	0	1.985405	-3.792082	0.800137

T-**DR2** at θ = 12 ° (C1-C2-C6 = C8-C7-C12 = 160 °)

Standard orientation:

Center Number	Atomic Number	Atomic Type	Coordinates (Angstroms) X	Y	Z
1	6	0	-1.778425	1.838156	0.022459
2	1	0	0.169527	2.763895	0.013719
3	6	0	-0.379279	1.830450	-0.091343
4	6	0	-1.778425	-0.593602	-0.261468
5	6	0	0.351400	0.622126	-0.232055
6	6	0	-2.465944	0.584990	-0.149948
7	6	0	-0.351400	-0.622126	-0.232055
8	6	0	1.778425	0.593602	-0.261468
9	1	0	-3.552689	0.580948	-0.117861
10	1	0	-0.169527	-2.763895	0.013719
11	1	0	-2.317324	-1.536268	-0.324586
12	6	0	2.465944	-0.584990	-0.149948
13	1	0	2.317324	1.536268	-0.324586
14	1	0	3.552689	-0.580948	-0.117861
15	6	0	1.778425	-1.838156	0.022459
16	6	0	0.379279	-1.830450	-0.091343
17	6	0	-2.471652	2.963377	0.540698
18	1	0	-3.544747	2.937354	0.698133
19	1	0	-1.950991	3.889554	0.760541
20	6	0	2.471652	-2.963377	0.540698
21	1	0	3.544747	-2.937354	0.698133
22	1	0	1.950991	-3.889554	0.760541

S-**DR2** at θ = 17 ° (C1-C2-C6 = C8-C7-C12 = 140 °)

Standard orientation:

Center Number	Atomic Number	Atomic Type	Coordinates (Angstroms) X	Y	Z
1	6	0	-1.858859	1.718219	0.086645
2	1	0	0.070236	2.750006	-0.040774
3	6	0	-0.420060	1.782712	-0.129879
4	6	0	-1.777068	-0.676653	-0.426028
5	6	0	0.325836	0.658214	-0.329704
6	6	0	-2.505280	0.446584	-0.248470
7	6	0	-0.325836	-0.658214	-0.329704
8	6	0	1.777068	0.676653	-0.426028
9	1	0	-3.591873	0.410563	-0.256054
10	1	0	-0.070236	-2.750006	-0.040774
11	1	0	-2.266634	-1.634645	-0.586532
12	6	0	2.505280	-0.446584	-0.248470
13	1	0	2.266634	1.634645	-0.586532
14	1	0	3.591873	-0.410563	-0.256054
15	6	0	1.858859	-1.718219	0.086645
16	6	0	0.420060	-1.782712	-0.129879
17	6	0	-2.505280	2.657646	0.822018
18	1	0	-3.559387	2.561731	1.066039
19	1	0	-1.999299	3.553732	1.169833
20	6	0	2.505280	-2.657646	0.822018
21	1	0	3.559387	-2.561731	1.066039
22	1	0	1.999299	-3.553732	1.169833

T-**DR2** at θ = 17 ° (C1-C2-C6 = C8-C7-C12 = 140 °)

Standard orientation:

Center Number	Atomic Number	Atomic Type	Coordinates (Angstroms) X	Y	Z
1	6	0	-1.811127	1.777856	0.038351
2	1	0	0.115429	2.750992	0.023660
3	6	0	-0.416858	1.815248	-0.132892
4	6	0	-1.764748	-0.631620	-0.387232
5	6	0	0.336707	0.630195	-0.343834
6	6	0	-2.476791	0.525347	-0.219309
7	6	0	-0.336707	-0.630195	-0.343834
8	6	0	1.764748	0.631620	-0.387232
9	1	0	-3.562611	0.494842	-0.171711
10	1	0	-0.115429	-2.750992	0.023660
11	1	0	-2.283356	-1.582912	-0.482842
12	6	0	2.476791	-0.525347	-0.219309
13	1	0	2.283356	1.582912	-0.482842
14	1	0	3.562611	-0.494842	-0.171711
15	6	0	1.811127	-1.777856	0.038351
16	6	0	0.416858	-1.815248	-0.132892
17	6	0	-2.476791	2.778226	0.793596
18	1	0	-3.533696	2.696033	1.024995
19	1	0	-1.958056	3.675973	1.113810
20	6	0	2.476791	-2.778226	0.793596
21	1	0	3.533696	-2.696033	1.024995
22	1	0	1.958056	-3.675973	1.113810

S-**DR2** θ = 26 ° (C1-C2-C6 = C8-C7-C12 = 120 °)

Standard orientation:

Center Number	Atomic Number	Atomic Type	Coordinates (Angstroms) X	Y	Z
1	6	0	-1.908250	1.600672	0.122600
2	1	0	-0.035720	2.739938	-0.048371
3	6	0	-0.484154	1.755204	-0.162083
4	6	0	-1.748811	-0.743313	-0.557770
5	6	0	0.299669	0.671937	-0.426154
6	6	0	-2.518239	0.340355	-0.322112
7	6	0	-0.299669	-0.671937	-0.426154
8	6	0	1.748811	0.743313	-0.557770
9	1	0	-3.602955	0.268798	-0.338444
10	1	0	0.035720	-2.739938	-0.048371
11	1	0	-2.199223	-1.708724	-0.777382
12	6	0	2.518239	-0.340355	-0.322112
13	1	0	2.199223	1.708724	-0.777382
14	1	0	3.602955	-0.268798	-0.338444
15	6	0	1.908250	-1.600672	0.122600
16	6	0	0.484154	-1.755204	-0.162083
17	6	0	-2.518239	2.366755	1.059946
18	1	0	-3.541810	2.177093	1.370980
19	1	0	-2.019346	3.222264	1.506647
20	6	0	2.518239	-2.366755	1.059946
21	1	0	3.541810	-2.177093	1.370980
22	1	0	2.019346	-3.222264	1.506647

T-**DR2** θ = 26 ° (C1-C2-C6 = C8-C7-C12 = 120 °)

Standard orientation:

Center Number	Atomic Number	Atomic Type	Coordinates (Angstroms) X	Y	Z
1	6	0	-1.866711	1.677981	0.059061
2	1	0	0.019715	2.731789	0.037311
3	6	0	-0.482449	1.789202	-0.169358
4	6	0	-1.738892	-0.697854	-0.506316
5	6	0	0.310873	0.643256	-0.448963
6	6	0	-2.492944	0.422206	-0.282893
7	6	0	-0.310873	-0.643256	-0.448963
8	6	0	1.738892	0.697854	-0.506316
9	1	0	-3.575939	0.346742	-0.221400
10	1	0	-0.019715	-2.731789	0.037311
11	1	0	-2.221337	-1.664099	-0.635546
12	6	0	2.492944	-0.422206	-0.282893
13	1	0	2.221337	1.664099	-0.635546
14	1	0	3.575939	-0.346742	-0.221400
15	6	0	1.866711	-1.677981	0.059061
16	6	0	0.482449	-1.789202	-0.169358
17	6	0	-2.492944	2.506887	1.025167
18	1	0	-3.523232	2.341219	1.323406
19	1	0	-1.981616	3.371609	1.436040
20	6	0	2.492944	-2.506887	1.025167
21	1	0	3.523232	-2.341219	1.323406
22	1	0	1.981616	-3.371609	1.436040

S-**DR3** at θ = 0 ° (C1-C5-C6 = C10-C6-C5 = 180 °)

Standard orientation:

Center Number	Atomic Number	Atomic Type	Coordinates (Angstroms) X	Y	Z
1	6	0	0.000000	3.597497	-0.268693
2	1	0	0.000000	3.333337	1.884037
3	6	0	0.000000	2.817047	0.926293
4	6	0	0.000000	1.499797	-1.540514
5	6	0	0.000000	1.427412	0.912478
6	6	0	0.000000	2.863282	-1.512371
7	6	0	0.000000	0.710946	-0.341649
8	6	0	0.000000	0.677977	2.143982
9	1	0	0.000000	3.425624	-2.442865
10	1	0	0.000000	-1.003220	-2.504765
11	1	0	0.000000	1.003220	-2.504765
12	6	0	0.000000	-0.677977	2.143982
13	1	0	0.000000	1.231857	3.079690
14	1	0	0.000000	-1.231857	3.079690
15	6	0	0.000000	-1.427412	0.912478
16	6	0	0.000000	-2.817047	0.926293
17	6	0	0.000000	-0.710946	-0.341649
18	6	0	0.000000	-3.597497	-0.268693
19	1	0	0.000000	-3.333337	1.884037
20	6	0	0.000000	-2.863282	-1.512371
21	1	0	0.000000	-3.425624	-2.442865
22	6	0	0.000000	-1.499797	-1.540514
23	6	0	0.000000	4.981919	-0.244144
24	1	0	0.000000	5.530158	0.692763
25	6	0	0.000000	-4.981919	-0.244144
26	1	0	0.000000	-5.562706	-1.161142
27	1	0	0.000000	5.562706	-1.161142
28	1	0	0.000000	-5.530158	0.692763

T-**DR3** at θ = 0 ° (C1-C5-C6 = C10-C6-C5 = 180 °)

Standard orientation:

Center Number	Atomic Number	Atomic Type	Coordinates (Angstroms) X	Y	Z
1	6	0	0.000000	3.599706	-0.264149
2	1	0	0.000000	3.344568	1.879235
3	6	0	0.000000	2.833904	0.918546
4	6	0	0.000000	1.504520	-1.532191
5	6	0	0.000000	1.425187	0.903165
6	6	0	0.000000	2.877152	-1.501707
7	6	0	0.000000	0.726338	-0.341809
8	6	0	0.000000	0.679799	2.131264
9	1	0	0.000000	3.438048	-2.433059
10	1	0	0.000000	-1.010232	-2.497801
11	1	0	0.000000	1.010232	-2.497801
12	6	0	0.000000	-0.679799	2.131264
13	1	0	0.000000	1.231664	3.068183
14	1	0	0.000000	-1.231664	3.068183
15	6	0	0.000000	-1.425187	0.903165
16	6	0	0.000000	-2.833904	0.918546
17	6	0	0.000000	-0.726338	-0.341809
18	6	0	0.000000	-3.599706	-0.264149
19	1	0	0.000000	-3.344568	1.879235
20	6	0	0.000000	-2.877152	-1.501707
21	1	0	0.000000	-3.438048	-2.433059
22	6	0	0.000000	-1.504520	-1.532191
23	6	0	0.000000	5.011446	-0.239408
24	1	0	0.000000	5.556070	0.698982
25	6	0	0.000000	-5.011446	-0.239408
26	1	0	0.000000	-5.589013	-1.157813

27	1	0	0.000000	5.589013	-1.157813
28	1	0	0.000000	-5.556070	0.698982

S-**DR3** at θ = 17 ° (C1-C5-C6 = C10-C6-C5 = 160 °)

Standard orientation:

Center Number	Atomic Number	Atomic Type	Coordinates (Angstroms) X	Y	Z
1	6	0	-0.298494	0.320260	3.469717
2	1	0	1.862739	0.335086	3.249741
3	6	0	0.921890	0.107842	2.752851
4	6	0	-1.505173	-0.493006	1.495471
5	6	0	0.940112	-0.266388	1.417897
6	6	0	-1.511146	-0.096717	2.798879
7	6	0	-0.300348	-0.496074	0.709766
8	6	0	2.179685	-0.304987	0.677452
9	1	0	-2.448340	-0.045350	3.347525
10	1	0	-2.448149	-0.751614	-1.025354
11	1	0	-2.448149	-0.751614	1.025354
12	6	0	2.179685	-0.304987	-0.677452
13	1	0	3.112907	-0.256832	1.233374
14	1	0	3.112907	-0.256832	-1.233374
15	6	0	0.940112	-0.266388	-1.417897
16	6	0	0.921890	0.107842	-2.752851
17	6	0	-0.300348	-0.496074	-0.709766
18	6	0	-0.298494	0.320260	-3.469717
19	1	0	1.862739	0.335086	-3.249741
20	6	0	-1.511146	-0.096717	-2.798879
21	1	0	-2.448340	-0.045350	-3.347525
22	6	0	-1.505173	-0.493006	-1.495471
23	6	0	-0.326950	0.947887	4.699182
24	1	0	0.585326	1.280139	5.184718
25	6	0	-0.326950	0.947887	-4.699182
26	1	0	-1.261935	1.125673	-5.221265
27	1	0	-1.261935	1.125673	5.221265
28	1	0	0.585326	1.280139	-5.184718

T-**DR3** at θ = 17 ° (C1-C5-C6 = C10-C6-C5 = 160 °)

Standard orientation:

Center Number	Atomic Number	Atomic Type	Coordinates (Angstroms) X	Y	Z
1	6	0	-0.291659	0.309165	3.476133
2	1	0	1.857049	0.336673	3.259145
3	6	0	0.912528	0.101890	2.772990
4	6	0	-1.497099	-0.484023	1.498091
5	6	0	0.928430	-0.277387	1.416191
6	6	0	-1.500446	-0.083206	2.811174
7	6	0	-0.299704	-0.510618	0.728327
8	6	0	2.163883	-0.290493	0.679471
9	1	0	-2.441037	-0.010675	3.351541
10	1	0	-2.445511	-0.718035	-1.025789
11	1	0	-2.445511	-0.718035	1.025789
12	6	0	2.163883	-0.290493	-0.679471
13	1	0	3.097086	-0.220644	1.233136
14	1	0	3.097086	-0.220644	-1.233136
15	6	0	0.928430	-0.277387	-1.416191
16	6	0	0.912528	0.101890	-2.772990
17	6	0	-0.299704	-0.510618	-0.728327
18	6	0	-0.291659	0.309165	-3.476133
19	1	0	1.857049	0.336673	-3.259145
20	6	0	-1.500446	-0.083206	-2.811174
21	1	0	-2.441037	-0.010675	-3.351541

22	6	0	-1.497099	-0.484023	-1.498091
23	6	0	-0.317955	0.940723	4.737776
24	1	0	0.597794	1.260813	5.223724
25	6	0	-0.317955	0.940723	-4.737776
26	1	0	-1.253248	1.115557	-5.259194
27	1	0	-1.253248	1.115557	5.259194
28	1	0	0.597794	1.260813	-5.223724

S-**DR3** at θ = 34 ° (C1-C5-C6 = C10-C6-C5 = 140 °)

Standard orientation:

Center Number	Atomic Number	Atomic Type	Coordinates (Angstroms) X	Y	Z
1	6	0	-0.448217	0.573442	3.087839
2	1	0	1.721429	0.855526	3.009320
3	6	0	0.880381	0.336610	2.554170
4	6	0	-1.306194	-1.066256	1.481820
5	6	0	1.065060	-0.360430	1.389868
6	6	0	-1.485858	-0.335757	2.607611
7	6	0	-0.090900	-0.925857	0.698329
8	6	0	2.334860	-0.335552	0.675129
9	1	0	-2.436856	-0.358678	3.134042
10	1	0	-2.119762	-1.675873	-1.100457
11	1	0	-2.119762	-1.675873	1.100457
12	6	0	2.334860	-0.335552	-0.675129
13	1	0	3.252150	-0.190646	1.240462
14	1	0	3.252150	-0.190646	-1.240462
15	6	0	1.065060	-0.360430	-1.389868
16	6	0	0.880381	0.336610	-2.554170
17	6	0	-0.090900	-0.925857	-0.698329
18	6	0	-0.448217	0.573442	-3.087839
19	1	0	1.721429	0.855526	-3.009320
20	6	0	-1.485858	-0.335757	-2.607611
21	1	0	-2.436856	-0.358678	-3.134042
22	6	0	-1.306194	-1.066256	-1.481820
23	6	0	-0.734262	1.645432	3.877557
24	1	0	0.038203	2.341883	4.189864
25	6	0	-0.734262	1.645432	-3.877557
26	1	0	-1.744387	1.837984	-4.226631
27	1	0	-1.744387	1.837984	4.226631
28	1	0	0.038203	2.341883	-4.189864

T-**DR3** at θ = 34 ° (C1-C5-C6 = C10-C6-C5 = 140 °)

Standard orientation:

Center Number	Atomic Number	Atomic Type	Coordinates (Angstroms) X	Y	Z
1	6	0	-0.428304	0.547981	3.114238
2	1	0	1.713400	0.845501	3.015162
3	6	0	0.864802	0.309776	2.595015
4	6	0	-1.300985	-1.038922	1.470606
5	6	0	1.047285	-0.404661	1.393684
6	6	0	-1.479556	-0.292028	2.606820
7	6	0	-0.078975	-0.973755	0.734286
8	6	0	2.298565	-0.300968	0.678700
9	1	0	-2.456014	-0.256136	3.083603
10	1	0	-2.149620	-1.577724	-1.061056
11	1	0	-2.149620	-1.577724	1.061056
12	6	0	2.298565	-0.300968	-0.678700
13	1	0	3.205661	-0.081298	1.236620
14	1	0	3.205661	-0.081298	-1.236620
15	6	0	1.047285	-0.404661	-1.393684
16	6	0	0.864802	0.309776	-2.595015

17	6	0	-0.078975	-0.973755	-0.734286
18	6	0	-0.428304	0.547981	-3.114238
19	1	0	1.713400	0.845501	-3.015162
20	6	0	-1.479556	-0.292028	-2.606820
21	1	0	-2.456014	-0.256136	-3.083603
22	6	0	-1.300985	-1.038922	-1.470606
23	6	0	-0.704145	1.640533	3.958426
24	1	0	0.086005	2.304413	4.293647
25	6	0	-0.704145	1.640533	-3.958426
26	1	0	-1.711553	1.837512	-4.310322
27	1	0	-1.711553	1.837512	4.310322
28	1	0	0.086005	2.304413	-4.293647

S-**DR3** at θ = 52 ° (C1-C5-C6 = C10-C6-C5 = 120 °)

Standard orientation:

Center Number	Atomic Number	Atomic Type	Coordinates (Angstroms) X	Y	Z
1	6	0	0.793823	-0.566514	2.505069
2	1	0	-1.142664	-1.613449	2.618166
3	6	0	-0.638380	-0.725143	2.243875
4	6	0	0.686482	1.638903	1.463489
5	6	0	-1.256131	0.085792	1.341423
6	6	0	1.305573	0.799954	2.320867
7	6	0	-0.449214	1.130078	0.693521
8	6	0	-2.513980	-0.281962	0.673819
9	1	0	2.230039	1.085500	2.816845
10	1	0	1.104252	2.616159	-1.237665
11	1	0	1.104252	2.616159	1.237665
12	6	0	-2.513980	-0.281962	-0.673819
13	1	0	-3.350537	-0.662444	1.254184
14	1	0	-3.350537	-0.662444	-1.254184
15	6	0	-1.256131	0.085792	-1.341423
16	6	0	-0.638380	-0.725143	-2.243875
17	6	0	-0.449214	1.130078	-0.693521
18	6	0	0.793823	-0.566514	-2.505069
19	1	0	-1.142664	-1.613449	-2.618166
20	6	0	1.305573	0.799954	-2.320867
21	1	0	2.230039	1.085500	-2.816845
22	6	0	0.686482	1.638903	-1.463489
23	6	0	1.613135	-1.628215	2.702405
24	1	0	1.224678	-2.640558	2.766520
25	6	0	1.613135	-1.628215	-2.702405
26	1	0	2.686379	-1.502566	-2.812862
27	1	0	2.686379	-1.502566	2.812862
28	1	0	1.224678	-2.640558	-2.766520

T-**DR3** at θ = 52 ° (C1-C5-C6 = C10-C6-C5 = 120 °)

Standard orientation:

Center Number	Atomic Number	Atomic Type	Coordinates (Angstroms) X	Y	Z
1	6	0	-0.769382	0.540372	2.552207
2	1	0	1.131139	1.591545	2.626575
3	6	0	0.623213	0.684386	2.306291
4	6	0	-0.669841	-1.621827	1.424325
5	6	0	1.258857	-0.143494	1.359243
6	6	0	-1.312514	-0.770951	2.282294
7	6	0	0.518661	-1.188245	0.746601
8	6	0	2.446920	0.352845	0.676648
9	1	0	-2.297111	-1.025276	2.667115
10	1	0	-1.164416	-2.539321	-1.119274
11	1	0	-1.164416	-2.539321	1.119274

12	6	0	2.446920	0.352845	-0.676648
13	1	0	3.221701	0.862024	1.244675
14	1	0	3.221701	0.862024	-1.244675
15	6	0	1.258857	-0.143494	-1.359243
16	6	0	0.623213	0.684386	-2.306291
17	6	0	0.518661	-1.188245	-0.746601
18	6	0	-0.769382	0.540372	-2.552207
19	1	0	1.131139	1.591545	-2.626575
20	6	0	-1.312514	-0.770951	-2.282294
21	1	0	-2.297111	-1.025276	-2.667115
22	6	0	-0.669841	-1.621827	-1.424325
23	6	0	-1.600425	1.639587	2.807789
24	1	0	-1.191131	2.637511	2.927680
25	6	0	-1.600425	1.639587	-2.807789
26	1	0	-2.673108	1.517484	-2.918492
27	1	0	-2.673108	1.517484	2.918492
28	1	0	-1.191131	2.637511	-2.927680

S-**DR4** at θ = 0 ° (C1-C5-C6 = C10-C13-C14 = 180 °)

Standard orientation:

Center Number	Atomic Number	Atomic Type	Coordinates (Angstroms) X	Y	Z
1	6	0	3.720393	0.654859	0.000000
2	1	0	2.490364	2.445770	0.000000
3	6	0	2.471114	1.357894	0.000000
4	6	0	2.471114	-1.458491	0.000000
5	6	0	1.246652	0.703023	0.000000
6	6	0	3.655838	-0.799881	0.000000
7	6	0	1.210869	-0.753004	0.000000
8	6	0	0.003970	1.405797	0.000000
9	1	0	4.592643	-1.351521	0.000000
10	1	0	-0.025989	-2.493953	0.000000
11	1	0	2.446399	-2.545934	0.000000
12	6	0	-1.210869	0.753004	0.000000
13	1	0	0.025989	2.493953	0.000000
14	6	0	-2.471114	1.458491	0.000000
15	6	0	-1.246652	-0.703023	0.000000
16	1	0	-2.490364	-2.445770	0.000000
17	6	0	-0.003970	-1.405797	0.000000
18	6	0	-3.655838	0.799881	0.000000
19	1	0	-2.446399	2.545934	0.000000
20	1	0	-4.592643	1.351521	0.000000
21	6	0	-3.720393	-0.654859	0.000000
22	6	0	-2.471114	-1.357894	0.000000
23	6	0	4.927078	1.314302	0.000000
24	1	0	5.867910	0.772648	0.000000
25	6	0	-4.927078	-1.314302	0.000000
26	1	0	-5.867910	-0.772648	0.000000
27	1	0	4.979279	2.398755	0.000000
28	1	0	-4.979279	-2.398755	0.000000

T-**DR4** at θ = 0 ° (C1-C5-C6 = C10-C13-C14 = 180 °)

Standard orientation:

Center Number	Atomic Number	Atomic Type	Coordinates (Angstroms) X	Y	Z
1	6	0	3.706485	0.711115	0.000000
2	1	0	2.481459	2.485746	0.000000
3	6	0	2.480770	1.397975	0.000000
4	6	0	2.480770	-1.414349	0.000000
5	6	0	1.229755	0.716833	0.000000

6	6	0	3.661266	-0.737949	0.000000
7	6	0	1.220136	-0.724910	0.000000
8	6	0	0.003218	1.401925	0.000000
9	1	0	4.603089	-1.281082	0.000000
10	1	0	-0.006460	-2.490240	0.000000
11	1	0	2.472071	-2.501991	0.000000
12	6	0	-1.220136	0.724910	0.000000
13	1	0	0.006460	2.490240	0.000000
14	6	0	-2.480770	1.414349	0.000000
15	6	0	-1.229755	-0.716833	0.000000
16	1	0	-2.481459	-2.485746	0.000000
17	6	0	-0.003218	-1.401925	0.000000
18	6	0	-3.661266	0.737949	0.000000
19	1	0	-2.472071	2.501991	0.000000
20	1	0	-4.603089	1.281082	0.000000
21	6	0	-3.706485	-0.711115	0.000000
22	6	0	-2.480770	-1.397975	0.000000
23	6	0	4.937786	1.388908	0.000000
24	1	0	5.878165	0.847687	0.000000
25	6	0	-4.937786	-1.388908	0.000000
26	1	0	-5.878165	-0.847687	0.000000
27	1	0	4.981496	2.473133	0.000000
28	1	0	-4.981496	-2.473133	0.000000

S-**DR4** at *θ* = 12 ° (C1-C5-C6 = C10-C13-C14 = 160 °)

Standard orientation:

Center Number	Atomic Number	Atomic Type	Coordinates (Angstroms) X	Y	Z
1	6	0	0.086707	3.709270	0.258569
2	1	0	2.041075	2.755041	0.232197
3	6	0	0.977646	2.599388	0.062643
4	6	0	-1.785705	2.248833	-0.363010
5	6	0	0.519590	1.326268	-0.235417
6	6	0	-1.318618	3.482762	-0.054445
7	6	0	-0.913089	1.095090	-0.387367
8	6	0	1.391481	0.193503	-0.291147
9	1	0	-1.998129	4.329186	0.007261
10	1	0	-2.465627	-0.366756	-0.263099
11	1	0	-2.846650	2.091379	-0.543990
12	6	0	0.913089	-1.095090	-0.387367
13	1	0	2.465627	0.366756	-0.263099
14	6	0	1.785705	-2.248833	-0.363010
15	6	0	-0.519590	-1.326268	-0.235417
16	1	0	-2.041075	-2.755041	0.232197
17	6	0	-1.391481	-0.193503	-0.291147
18	6	0	1.318618	-3.482762	-0.054445
19	1	0	2.846650	-2.091379	-0.543990
20	1	0	1.998129	-4.329186	0.007261
21	6	0	-0.086707	-3.709270	0.258569
22	6	0	-0.977646	-2.599388	0.062643
23	6	0	0.519590	4.904328	0.775483
24	1	0	-0.164916	5.730466	0.941577
25	6	0	-0.519590	-4.904328	0.775483
26	1	0	0.164916	-5.730466	0.941577
27	1	0	1.561821	5.064362	1.034188
28	1	0	-1.561821	-5.064362	1.034188

T-**DR4** at θ = 12 ° (C1-C5-C6 = C10-C13-C14 = 160 °)

Standard orientation:

Center Number	Atomic Number	Atomic Type	Coordinates (Angstroms) X	Y	Z
1	6	0	0.081455	3.706958	0.249689
2	1	0	2.034651	2.792532	0.206774
3	6	0	0.970266	2.637576	0.044499
4	6	0	-1.786274	2.229143	-0.339152
5	6	0	0.511484	1.322058	-0.254271
6	6	0	-1.322345	3.469861	-0.029168
7	6	0	-0.905743	1.092407	-0.391940
8	6	0	1.382183	0.219274	-0.289177
9	1	0	-2.011599	4.306310	0.056287
10	1	0	-2.455176	-0.391113	-0.230989
11	1	0	-2.850417	2.064434	-0.492539
12	6	0	0.905743	-1.092407	-0.391940
13	1	0	2.455176	0.391113	-0.230989
14	6	0	1.786274	-2.229143	-0.339152
15	6	0	-0.511484	-1.322058	-0.254271
16	1	0	-2.034651	-2.792532	0.206774
17	6	0	-1.382183	-0.219274	-0.289177
18	6	0	1.322345	-3.469861	-0.029168
19	1	0	2.850417	-2.064434	-0.492539
20	1	0	2.011599	-4.306310	0.056287
21	6	0	-0.081455	-3.706958	0.249689
22	6	0	-0.970266	-2.637576	0.044499
23	6	0	0.511484	4.941667	0.764053
24	1	0	-0.186406	5.755146	0.932908
25	6	0	-0.511484	-4.941667	0.764053
26	1	0	0.186406	-5.755146	0.932908
27	1	0	1.555760	5.118067	1.000361
28	1	0	-1.555760	-5.118067	1.000361

S-**DR4** at θ = 24 ° (C1-C5-C6 = C10-C13-C14 = 140 °)

Standard orientation:

Center Number	Atomic Number	Atomic Type	Coordinates (Angstroms) X	Y	Z
1	6	0	0.286225	3.495835	0.516746
2	1	0	2.177837	2.399324	0.484781
3	6	0	1.141848	2.381980	0.152355
4	6	0	-1.544061	2.427216	-0.717471
5	6	0	0.640028	1.250004	-0.435930
6	6	0	-1.036031	3.522958	-0.111900
7	6	0	-0.796048	1.177664	-0.744875
8	6	0	1.407840	0.034548	-0.564906
9	1	0	-1.631041	4.427704	-0.013540
10	1	0	-2.493699	-0.101458	-0.534384
11	1	0	-2.560343	2.428498	-1.104879
12	6	0	0.796048	-1.177664	-0.744875
13	1	0	2.493699	0.101458	-0.534384
14	6	0	1.544061	-2.427216	-0.717471
15	6	0	-0.640028	-1.250004	-0.435930
16	1	0	-2.177837	-2.399324	0.484781
17	6	0	-1.407840	-0.034548	-0.564906
18	6	0	1.036031	-3.522958	-0.111900
19	1	0	2.560343	-2.428498	-1.104879
20	1	0	1.631041	-4.427704	-0.013540
21	6	0	-0.286225	-3.495835	0.516746
22	6	0	-1.141848	-2.381980	0.152355
23	6	0	0.640028	4.393504	1.475845
24	1	0	-0.027347	5.196596	1.773955
25	6	0	-0.640028	-4.393504	1.475845

26	1	0	0.027347	-5.196596	1.773955
27	1	0	1.603035	4.340287	1.974875
28	1	0	-1.603035	-4.340287	1.974875

T-**DR4** at θ = 24 ° (C1-C5-C6 = C10-C13-C14 = 140 °)

Standard orientation:

Center Number	Atomic Number	Atomic Type	Coordinates (Angstroms) X	Y	Z
1	6	0	0.243315	3.505693	0.489377
2	1	0	2.148722	2.490954	0.413206
3	6	0	1.107298	2.464070	0.099767
4	6	0	-1.601889	2.364200	-0.652379
5	6	0	0.607760	1.264073	-0.487456
6	6	0	-1.107298	3.476171	-0.046877
7	6	0	-0.807588	1.162080	-0.757108
8	6	0	1.390748	0.096854	-0.553878
9	1	0	-1.745050	4.341814	0.115341
10	1	0	-2.469737	-0.172739	-0.436520
11	1	0	-2.644201	2.325756	-0.960925
12	6	0	0.807588	-1.162080	-0.757108
13	1	0	2.469737	0.172739	-0.436520
14	6	0	1.601889	-2.364200	-0.652379
15	6	0	-0.607760	-1.264073	-0.487456
16	1	0	-2.148722	-2.490954	0.413206
17	6	0	-1.390748	-0.096854	-0.553878
18	6	0	1.107298	-3.476171	-0.046877
19	1	0	2.644201	-2.325756	-0.960925
20	1	0	1.745050	-4.341814	0.115341
21	6	0	-0.243315	-3.505693	0.489377
22	6	0	-1.107298	-2.464070	0.099767
23	6	0	0.614469	4.462341	1.445630
24	1	0	-0.073358	5.241069	1.758729
25	6	0	-0.614469	-4.462341	1.445630
26	1	0	0.073358	-5.241069	1.758729
27	1	0	1.605481	4.458948	1.887710
28	1	0	-1.605481	-4.458948	1.887710

S-**DR4** at θ = 35 ° (C1-C5-C6 = C10-C13-C14 = 120 °)

Standard orientation:

Center Number	Atomic Number	Atomic Type	Coordinates (Angstroms) X	Y	Z
1	6	0	0.373659	3.163706	0.743594
2	1	0	2.207121	1.951821	0.736539
3	6	0	1.239135	2.091723	0.259602
4	6	0	-1.239135	2.596405	-1.005618
5	6	0	0.757193	1.140189	-0.591403
6	6	0	-0.755995	3.505280	-0.133961
7	6	0	-0.649555	1.253677	-1.044004
8	6	0	1.405660	-0.143819	-0.787651
9	1	0	-1.243000	4.468603	-0.002108
10	1	0	-2.492008	0.199043	-0.753783
11	1	0	-2.134735	2.797086	-1.588824
12	6	0	0.649555	-1.253677	-1.044004
13	1	0	2.492008	-0.199043	-0.753783
14	6	0	1.239135	-2.596405	-1.005618
15	6	0	-0.757193	-1.140189	-0.591403
16	1	0	-2.207121	-1.951821	0.736539
17	6	0	-1.405660	0.143819	-0.787651
18	6	0	0.755995	-3.505280	-0.133961
19	1	0	2.134735	-2.797086	-1.588824
20	1	0	1.243000	-4.468603	-0.002108
21	6	0	-0.373659	-3.163706	0.743594

22	6	0	-1.239135	-2.091723	0.259602
23	6	0	0.499740	3.681707	1.990614
24	1	0	-0.192287	4.429785	2.366401
25	6	0	-0.499740	-3.681707	1.990614
26	1	0	0.192287	-4.429785	2.366401
27	1	0	1.301948	3.373304	2.654734
28	1	0	-1.301948	-3.373304	2.654734

T-**DR4** at *θ* = 35 ° (C1-C5-C6 = C10-C13-C14 = 120 °)

Standard orientation:

Center Number	Atomic Number	Atomic Type	Coordinates (Angstroms) X	Y	Z
1	6	0	0.312424	3.190236	0.707525
2	1	0	2.181829	2.100000	0.614460
3	6	0	1.195562	2.224769	0.172364
4	6	0	-1.384625	2.497294	-0.915895
5	6	0	0.704692	1.187719	-0.679391
6	6	0	-0.916030	3.425022	-0.043688
7	6	0	-0.688936	1.229132	-1.068749
8	6	0	1.384625	-0.043895	-0.769910
9	1	0	-1.499481	4.314929	0.180960
10	1	0	-2.456996	0.079786	-0.592393
11	1	0	-2.360310	2.620914	-1.380384
12	6	0	0.688936	-1.229132	-1.068749
13	1	0	2.456996	-0.079786	-0.592393
14	6	0	1.384625	-2.497294	-0.915895
15	6	0	-0.704692	-1.187719	-0.679391
16	1	0	-2.181829	-2.100000	0.614460
17	6	0	-1.384625	0.043895	-0.769910
18	6	0	0.916030	-3.425022	-0.043688
19	1	0	2.360310	-2.620914	-1.380384
20	1	0	1.499481	-4.314929	0.180960
21	6	0	-0.312424	-3.190236	0.707525
22	6	0	-1.195562	-2.224769	0.172364
23	6	0	0.503443	3.759382	1.969858
24	1	0	-0.210345	4.465162	2.382681
25	6	0	-0.503443	-3.759382	1.969858
26	1	0	0.210345	-4.465162	2.382681
27	1	0	1.383723	3.530531	2.561987
28	1	0	-1.383723	-3.530531	2.561987
